# Effectiveness of decision aids for female *BRCA1* and *BRCA2* mutation carriers: a systematic review

**DOI:** 10.1186/s12911-019-0872-2

**Published:** 2019-08-01

**Authors:** Lisa Krassuski, Vera Vennedey, Stephanie Stock, Sibylle Kautz-Freimuth

**Affiliations:** Institute for Health Economics and Clinical Epidemiology, The University Hospital of Cologne (AöR), Gleueler Straße 176-178, 50935 Cologne, Germany

**Keywords:** *BRCA1* and *BRCA2*, Female *BRCA1* and *BRCA2* mutation carriers, Familial breast cancer, Familial ovarian cancer, Hereditary breast and ovarian cancer, HBOC, Decision aid, Decision-making

## Abstract

**Background:**

Female *BRCA1* and *BRCA2* mutation carriers have an increased lifetime risk of developing breast and/or ovarian cancer. Hence, they face the difficult decision of choosing a preventive strategy such as risk-reducing surgeries or intensified breast screening. To help these women during their decision process, several patient decision aids (DA) were developed and evaluated in the last 15 years. Until now, there is no conclusive evidence on the effectiveness of these DA. This study aims 1) to provide the first systematic literature review about DA addressing preventive strategy decisions for female *BRCA1* and *BRCA2* mutation carriers, 2) to analyze the quality of the existing evidence, 3) to evaluate the effects of DA on decision and information related outcomes, on the actual choice for preventive measure and on health outcomes.

**Methods:**

A systematic literature review was conducted using six electronic databases (inclusion criteria: DA addressing preventive strategies, female *BRCA1* and *BRCA2* mutation carriers, 18 to 75 years, knowledge of test result). The quality of the included randomized controlled trials (RCT) was evaluated with the Cochrane Collaboration’s risk of bias tool. The quality of included one-group pretest-posttest design studies was evaluated with the ROBINS-I tool. Outcomes of included studies were extracted and qualitatively summarized.

**Results:**

A total of 2093 records were identified. Six studies were included for further evaluation (5 RCT, 1 one-group pretest-posttest design study). One RCT was formally included, but data presentation did not allow for further analyses. The risk of bias was high in three RCT and unclear in one RCT. The risk of bias in the one-group pretest-posttest study was serious. The outcome assessment showed that the main advantages of DA are linked to the actual decision process: Female *BRCA1* and *BRCA2* mutation carriers using a DA had less decisional conflict, were more likely to reach a decision and were more satisfied with their decision.

**Conclusions:**

Decision aids can support female *BRCA1* and *BRCA2* mutation carriers during their decision process by significantly improving decision related outcomes. More high-quality evidence is needed to evaluate possible effects on information related outcomes, health outcomes and the actual choice for preventive measures.

**Electronic supplementary material:**

The online version of this article (10.1186/s12911-019-0872-2) contains supplementary material, which is available to authorized users.

## Background

Approximately 0.1 to 0.3% of all women carry a mutation in one of the so-called breast cancer genes *BRCA1* and *BRCA2* [1–5]. These women have an increased lifetime risk of developing breast (BC) and ovarian cancer (OC). According to population-based studies, the average cumulative risks in *BRCA1* mutation carriers by age 80 years are 72% for BC and 44% for OC. The corresponding estimates for *BRCA2* are 69 and 17% [6].

Genetic testing and counselling for a *BRCA1* and *BRCA2* mutation is strongly recommended for women with a family history or a personal history of BC and/or OC, which is potentially associated with hereditary mutations [7]. A positive genetic test result is followed by a series of questions and difficult, far-reaching decisions. Unaffected mutation carriers have to determine how they want to manage the elevated risk of developing cancer, considering their individual life situation and personal values. Mutation carriers with a personal history of BC and/or OC confront an even more complicated decision-making process: A woman with unilateral BC has to consider different competing risks when taking a decision, such as the risk of developing contralateral cancer, the risk of an ipsilateral relapse, the risk of developing OC, and the risks arising from the primary cancer disease.

Current strategies for female *BRCA1* and *BRCA2* mutation carriers to manage the elevated cancer risk include an intensified breast screening program (including magnetic resonance imaging, breast ultrasound, mammography, and breast palpation by a physician) as well as risk-reducing surgeries [7]. To date there is no effective OC screening program [8, 9]. For some women with a *BRCA1* and *BRCA2* mutation, another strategy to reduce the elevated cancer risk is chemoprevention (e.g. tamoxifen, aromatase inhibitors) [10, 11]. Furthermore, some studies indicate that the cancer risk of *BRCA1* and *BRCA2* mutation carriers might be reduced by a healthy lifestyle (e.g. no smoking, physical activity) [12, 13].

Surgical options include risk-reducing bilateral salpingo-oophorectomy (RR-BSO), risk-reducing bilateral mastectomy (RR-BM) and risk-reducing contralateral mastectomy (RR-CM). The latter is an option for mutation carriers after unilateral BC. The decision for a risk-reducing mastectomy (RR-M) is again followed by a series of upcoming questions, such as considering the surgical technique (e.g. complete, skin sparing or nipple sparing mastectomy) and having a breast reconstruction or not. The decision for a RR-BSO is followed by the question of whether receiving hormone replacement therapy (HRT) and, when indicated, which type of HRT should be chosen. Before choosing RR-BSO family planning should be completed.

In such complex situations, which require weighing up advantages and disadvantages of options, patient decision aids (DA) might be useful to support individual decision making. DA are tools for people seeking advice. The International Patient Decision Aid Standards (IPDAS) Collaboration defines them as “tools designed to help people participate in decision making about health care options. They provide information on the options and help patients clarify and communicate the personal value they associate with different features of the options” [14]. DA are generally used for complex decisions: 1) When there is more than one adequate option, 2) when no option has a clear advantage regarding health outcomes, 3) when each option has benefits, harms and uncertainties that the consumer may value differently, and – in some cases – 4) when the scientific evidence about options is limited [14, 15]. Complex decisions can be choices about medical screening, treatment or preventive options. The aim of DA is to reduce the consumers’ uncertainties and confusion, as well as to improve the quality of decisions – or as O’Connor states: “increase the likelihood that consumers will make ‘effective’ decisions” [16]. An “effective” decision is a decision that is informed, consistent with personal values, and acted upon [16].

Since a positive result of a *BRCA1* and *BRCA2* gene test is followed by a series of questions and difficult decisions, the need for adequate counseling is high. In this situation the use of a DA might be valuable during the decision-making process. A previous systematic literature review has shown that DA concerning different health decisions when compared to usual care, can improve the consumers’ knowledge and reduce their decisional conflict [17].

In the last 15 years several DA for female *BRCA1* and *BRCA2* mutation carriers were developed. Although some were evaluated in randomized controlled trials (RCT) or qualitative studies, to date, there is no systematic literature review about the effectiveness of DA for female *BRCA1* and *BRCA2* mutation carriers.

The aim of this review is to provide the first systematic literature review about the effectiveness of DA for female *BRCA1* and *BRCA2* mutation carriers, to analyze the quality of the existing evidence and to evaluate the effects of DA on decision related outcomes, information related outcomes, actual choice for preventive measure and health outcomes.

## Methods

### Literature search

The electronic databases MEDLINE, Embase, PsycINFO, CINAHL, ERIC, and Cochrane Database of Systematic Reviews were searched. We selected these databases since they focus on health, nursing and psychology publications. The search strategy included two categories of search terms: decision-making/decision aid and *BRCA1/2*. The search strategy was tailored to the requirements of each individual database (see Additional file 1). Whenever feasible, we followed the PRISMA guidelines [18]. Throughout the literature search we used the definition of DA provided by the IPDAS Collaboration [14, 19]. The final database research was performed February 5th, 2019.

### Inclusion and exclusion criteria

We included original studies evaluating the effectiveness of DA for women aged 18 to 75 years, who were tested positive for a *BRCA1* or *BRCA2* mutation and know their genetic test result. Furthermore, we only included DA addressing preventive strategy decisions. Publications were excluded if they did not meet the inclusion criteria or if they addressed the question of whether to undergo genetic testing. We also excluded studies regarding the development, the structure, and the implementation of DA for female *BRCA1* and *BRCA2* mutation carriers as well as studies evaluating other types of decision support such as decision coaching. There were no language restrictions. Publications in languages other than German, English, French and Spanish were translated by native speakers. A restriction to specific study designs was not implemented, since we wanted to give an overview of the whole existing evidence. There were no restrictions regarding the year of publication or the publication status.

### Paper coding

After removing the duplicate results, titles and abstracts were screened according to the eligibility criteria independently by two reviewers (LK, SKF). They were rejected if the reviewers determined from the title or abstract that the study did not meet the inclusion criteria. After this screening process, full texts were retrieved and further assessed for eligibility independently by two reviewers. Any disagreement was solved by discussion among the reviewers.

### Quality of included studies

The quality of RCT was evaluated using the Cochrane Collaborations’ risk of bias tool [20]. This tool uses seven criteria to measure quality: 1) random sequence generation, 2) allocation concealment, 3) blinding of participants and personnel, 4) blinding of outcome assessment, 5) incomplete outcome data, 6) selective reporting and 7) other sources of bias [20]. Each criterion was judged to have a low, high or unclear risk of bias. If one criterion of a study was considered as “high risk”, the study was classified as having a high risk of bias overall. The evaluation of study quality was performed independently by two reviewers (LK, SKF). A third reviewer (VV) was consulted in case of disagreement of item ratings.

The quality of the one-group pretest-posttest design study was evaluated with the Risk Of Bias In Non-randomised Studies - of Interventions (ROBINS-I) assessment tool [21], which is recommended for quality assessment of non-randomised studies of interventions. This tool uses six bias domains to measure quality: bias 1) due to confounding, 2) due to deviations from intended interventions, 3) due to missing data, 4) in selection of participants into the study, 5) in classification of interventions and 6) in selection of the reported result. Each domain was judged to have a low, moderate, serious or critical risk of bias. If one domain of a study was considered as “critical”, the study was classified as having a critical bias overall. That means that the study should not be included in a synthesis. Only if all the domains would be rated as having a low risk of bias, the study receives an overall rating of “low”. The evaluation of the studies was performed independently by three reviewers (LK, SKF, VV). In case of disagreement of item ratings, the three reviewers discussed until they would reach accordance.

### Data extraction and management

One reviewer (LK) extracted the study characteristics and outcome data from the included studies (Tables 1, 3, 4). Two reviewers (SKF, VV) compared the findings independently. We contacted authors to obtain missing data. We evaluated the effects of the DA based on the outcomes used in the trials. Hereby we categorized the outcomes in four categories: 1) decision related outcomes, 2) information related outcomes, 3) actual choice for preventive measure and 4) health outcomes. Due to the heterogeneity of the trials in study design, follow-up periods, and outcomes that hindered a meta-analysis, data were synthesized qualitatively.

**Table 1:** Characteristics of included studies

RCT - Parallel group	Author	Year of publication	Country	Time of recruitment	Inclusion criteria	No. of subjects randomized	Control: Comparator	Control: No. of subjects	Intervention	Intervention: No. of subjects
Individualized survival curves improve satisfaction with cancer risk management decisions in women with *BRCA1/2* mutations [22]	Armstrong	2005	USA	2000–2003	*BRCA1-* and *BRCA2-*positive women, with or without BC (no OC, no BC with metastases, no RR-BM + RR-BO)	32	Educational booklet	13	Educational booklet + binder with comprehension exercise, individualized survival curves and individualized BC incidence curves	14
Randomized trial of a decision aid for *BRCA1/BRCA2* mutation carriers: impact on measures of decision making and satisfaction [23]	Schwartz	2009	USA	2001–2005	*BRCA1*- and *BRCA2*-positive women, with or without BC/OC (no BC with metastases, no OC with metastases, no RR-BM)	214	Usual care	114	Usual care + interactive CD-ROM with information about BC and risk management options, tailored BC and OC risk graphs and an interactive decision task	100
Longitudinal changes in patient distress following interactive decision aid use among *BRCA1/2* carriers: a randomized trial [24]	Hooker	2011	USA	2001–2005	*BRCA1-* and *BRCA2-*positive women, with or without BC/OC (no BC with metastases, no OC with metastases, no RR-BM)	214	Usual care	114	Usual care + interactive CD-ROM with information about BC and risk management options, tailored breast and ovarian cancer risk graphs and an interactive decision task	100
Effect of decision aid for breast cancer prevention on decisional conflict in women with a *BRCA1* or *BRCA2* mutation: a multisite, randomized, controlled trial [25]	Metcalfe	2017	Canada	2008–2011	*BRCA1-* and *BRCA2-*positive women, without cancer (no RR-M, no RR-O, no tamoxifen)	150	Usual care	74	Usual care + booklet with information about BC risks, BC preventive options, guidelines, studies and a possibility to compare the options	76
RCT - Cross-over trial	Author	Year of publication	Country	Time of recruitement	Inclusion criteria	No. of subjects randomized	Control: Comparator	Group 1: No. of subjects	Intervention	Group 2: No. of subjects
Randomised trial of a decision aid and its timing for women being tested for a *BRCA1/2* mutation [26]	Van Roosmalen	2004	NL	1999–2001	*BRCA1-* and *BRCA2-*positive women, with or without BC/OC (no distant metastases, no RR-BM + RR-BO; no chemotherapy, radiotherapy or BC/OC surgery 1 month before blood sampling)	384	Usual care	T2 (before gen. Testing, gets DA): 184 T3 (positive test result): 47	Usual care + Brochure with information about treatment options + 45 min. Video with interviews mutation carriers	T2 (before gen. Testing, gets no DA): 184 T3 (positive test result, gets DA): 42
One-Group Pretest-Posttest Design	Author	Year of publication	Country	Time of recruitement	Inclusion criteria	No. of subjects willing to participate	Subjects completing the pre-test questionnaire	Intervention		Subjects completing the post-test questionnaire
Development and testing of a decision aid for breast cancer prevention for women with a *BRCA1* or *BRCA2* mutation [27]	Metcalfe	2007	Canada	Not specified	*BRCA1-* and *BRCA2-*positive women, without BC/OC	21	21	Brochure with information about options and outcomes, risks and benefits, a valuing exercise and suggestions for follow-up discussions with their practitioner		20

*RCT* randomized controlled trial, *NL* Netherlands, *CD-ROM* Compact Disc Read-Only Memory, *No.* number, *DA* decision aid, *BRCA1* breast cancer gene 1, *BRCA2* breast cancer gene 2, *OC* ovarian cancer, *BC* breast cancer, *RR-BM* risk-reducing bilateral mastectomy, *RR-M* risk-reducing mastectomy, *RR-BO* risk-reducing bilateral oophorectomy, *RR-O* risk-reducing oophorectomy, *T2* 4 weeks after blood sampling, *T3* 2 weeks after positive test result

## Results

### Search results

**Fig. 1:**
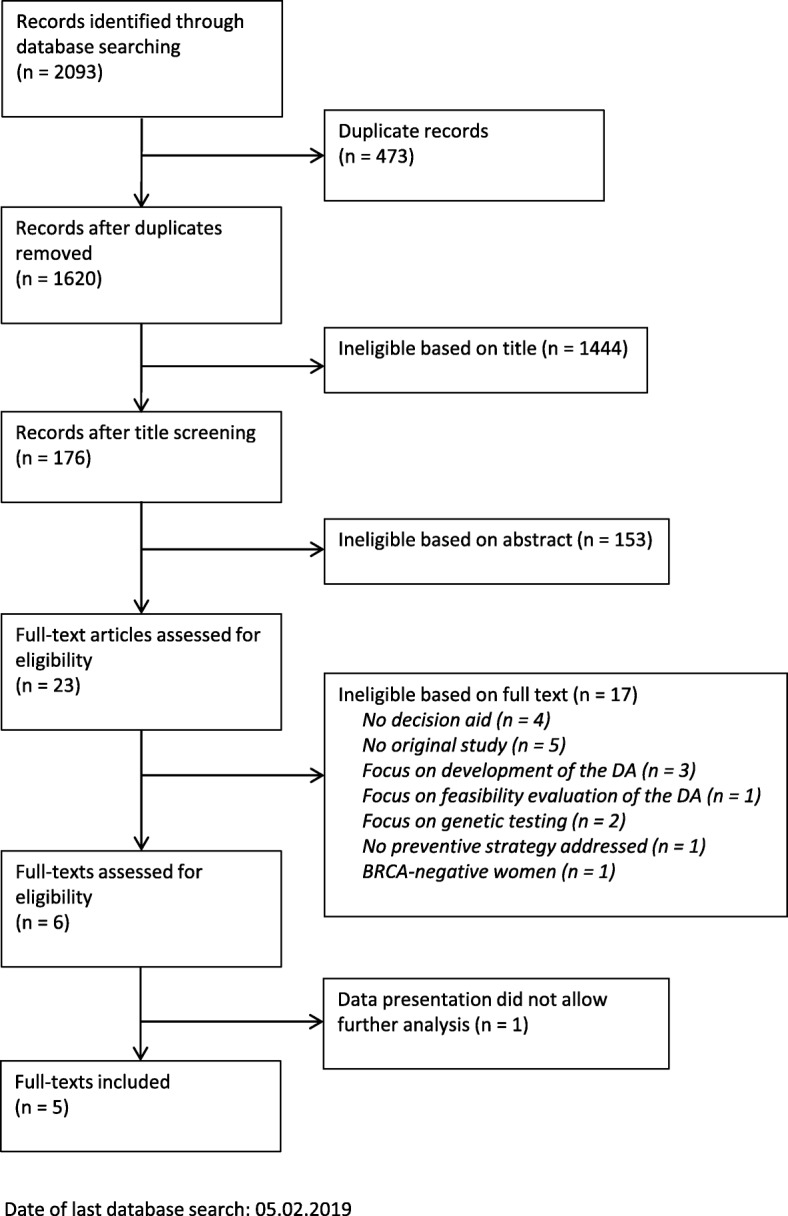
Flow chart of search strategy and study selection, according to the PRISMA guidelines

As shown in Fig. 1, a total of 2093 records were identified through database search. Of these records, six full-text studies were included in this review.

The main characteristics of the six included studies are shown in Table 1. Five studies are RCT, four of them having a parallel group design [22–25], one conducted as a randomized cross-over trial [26]. One study is a quasi-experiment with a one-group pretest-posttest design [27]. All six studies reported various effects of the DA on decision and information related outcomes, health related outcomes, and actual choice for preventive measure. One study [26] provided outcome data of female *BRCA1* and *BRCA2* mutation carriers at a point of time, when the women did not know their *BRCA1* or *BRCA2* test result yet. Despite contacting the authors and receiving more data, we still could not include the study, since the data set provided by the authors did not allow for tracing back the separate study arm in the form needed for our review.

### Quality of included studies

The quality analysis of the included RCT [22–25] showed that three studies were at high risk of bias [23–25] and one was of unclear risk of bias [22] (Table 2). All analyzed RCT adequately randomized patients and had no selective outcome reporting. Only one study reported adequate allocation concealment [22]. All RCT had deficits concerning the blinding of participants, personnel, and/or outcome assessors. A detailed description of the quality assessment of the RCT is provided in Additional file 2.

**Table 2:** Quality analysis of RCT according to the Cochrane Collaboration’s risk of bias tool [20]

Publication	Armstrong (2005) [22]	Schwartz (2009) [23]	Hooker (2011) [24]	Metcalfe (2017) [25]
Criterion				
Adequate sequence generation	+	+	+	+
Adequate allocation concealment	+	?	?	?
Blinding of participants and personnel	(−)^b^	(−)^c^	(−)^c^	–
Blinding of outcome assessors	+	-^a^	-^a^	-^a, d^
No incomplete outcome data	?	?	?	?
No selective outcome reporting	+	+	+	+
No other sources of bias	+	+	+	+
RISK OF BIAS	Unclear	High	High	High

+ Met criterion;? Unclear; − Unmet criterion; RCT: randomized controlled trial

^a^Patient-Reported Outcome Measures (PROMs) with unblinded patients

^b^patients and outcome assessors blinded, staff not blinded

^c^patients not blinded, blinding of staff not or not completely mentioned

^d^research assistant blinded but likely that blinding has been broken

The one-group pretest-posttest design study [27] was rated as having a serious risk of bias overall. There were serious deficits in the domains confounding and measurement of outcomes, the article did not provide sufficient information to judge the domain missing data. The bias in the other domains was rated as “low”. A detailed description of the quality assessment of the one-group pretest-posttest design study is provided in Additional file 3.

### Effects of the decision aids

The qualitative synthesis of the effectiveness was performed for all included six studies. A variety of outcomes were used to evaluate the effects of the DA. A summary of the outcomes, respective instruments as well as the corresponding main effects of the DA is provided in Tables 3 and 4.

**Table 3:** Outcomes, instruments used, and effects of decisions aids evaluated in the included RCT

Outcomes	Instruments used for assessment	RCT using the instrument	a) Score (S.D.) or [range] b) Regression analysis	*p*-value	Main results
Decision related outcomes					
Decisional conflict	Decisional Conflict Scale^a^	Schwartz 2009 [23]	b) Intervention, subjects were undecided at randomization: B − 0.35, z − 3.6	< 0.001	Significant decreases in decisional conflict in initially undecided women in the DA group.
			b) Intervention, subjects were decided at randomization: B − 0.10, z − 0.98	0.33	
		Metcalfe 2017 [25]	a) 3 month: Intervention 25.6 (13.2), Control 26.8 (12.6)	0.59	No significant effect.
			a) 6 month: Intervention 24.8 (13.8), Control 24.7 (12.8)	0.96	
			a) 12 month: Intervention 21.5 (13.7), Control 21.0 (12.3)	0.81	
Satisfaction with decision	Variation of Decisional Conflict Scale/Satisfaction With Decision Scale^c^	Armstrong 2005 [22]	a) Intervention 31.2, Control 26.2	0.04	Significantly higher decision satisfaction in the DA group.
	Satisfaction With Decision Scale^a^	Schwartz 2009 [23]	b) Intervention, subjects were undecided at randomization: B 0.27, z 3.1	0.002	Significant increase in satisfaction with decision in initially undecided women in the DA group.
			b) Intervention, subjects were decided at randomization: B − 0.07, z − 0.7	0.48	
Strenght of treatment preference	15-point scale^c^	Metcalfe 2017 [25]	a) Subjects reporting „undecided "(score 6–10):		No significant effect.
			RR-M:		
			3 month: Intervention 19, Control 15	0.52	
			6 month: Intervention 12, Control 15	0.47	
			12 month: Intervention 10, Control 15	0.81	
			RR-O:		
			3 month: Intervention 8, Control 2	0.05	
			6 month: Intervention 4, Control 7	0.33	
			12 month: Intervention 6, Control 7	0.66	
			Tamoxifen:		
			3 month: Intervention 15, Control 15	0.89	
			6 month: Intervention 10, Control 12	0.57	
			12 month: Intervention 10, Control 6	0.35	
	Final decision vs. No final decision	Schwartz 2009 [23]	b) Intervention, subjects were undecided at randomization: OR 3.09, 95% CI 1.62, 5.90	< 0.001	Significantly increased likelihood to reach a management decision in initially undecided women in the DA group.
			b) Intervention, subjects were decided at randomization: OR 0.56, 95% CI 0.24, 1.29	0.17	
		Hooker 2011 [24]	No data are presented.		No data are presented.
Information related outcomes					
Risk perception	Knowledge questionnaire (see also Metcalfe 2007)^c^	Metcalfe 2017 [25]	a) 3 month: Intervention 89.9 (9.4), Control 89.9 (9.8)	0.98	No significant effect.
			6 month: Intervention 90.1 (10.4), Control 89.7 (12.4)	0.55	
			12 month: Intervention 92.0 (10.3), Control 91.6 (10.2)	0.84	
	OC risk, mutation carriers^d^	Armstrong 2005 [22]	a) Intervention 54.0 [0–90)], Control 42.3 [0–80)]	0.54	No significant effect.
	BC, risk after RR-M, mutation carriers^d^	Armstrong 2005 [22]	a) Intervention 15.0 [0–25)], Control 10.3 [0–50]	0.56	No significant effect.
	BC, risk after RR-O, mutation carriers^d^	Armstrong 2005 [22]	a) Intervention 40.3 [0–80], Control 23.3 [0–80]	0.20	No significant effect.
	BC, risk with Tamoxifen, mutation carriers^d^	Armstrong 2005 [22]	a) Intervention 11.2 [0–60], Control 9.2 [0–40]	0.26	No significant effect.
	BC, risk with HRT after menopause, mutation carriers^d^	Armstrong 2005 [22]	a) Intervention 49.5 [0–90], Control 18.8 [0–45]	0.13	No significant effect.
	BC, risk with Raloxifene after menopause, mutation carriers^d^	Armstrong 2005 [22]	a) Intervention 42.5 [0–75], Control 12.5 [0–30]	0.08	No significant effect.
	BC, risk with mammography, mutation carriers^d^	Armstrong 2005 [22]	a) Intervention 63.8 [0–90], Control 41.7 [0–80]	0.12	No significant effect.
	OC, risk after RR-O, mutation carriers^d^	Armstrong 2005 [22]	a) Intervention 6.7 [0–60], Control 6.5 [0–50]	0.65	No significant effect.
Actual treatment choice	RR-M vs. No RR-M	Schwartz 2009 [23]	b) 0–12 month, subjects obtaining RR-M: Intervention 18, Control 15, χ2 (df = 1, *N* = 214) = 0.96	0.33	No difference in DA or control group in having a RR-M or not, but impact of the DA in timing of the RR-M (control: early after testing; DA: 6–12 month after testing).
			b) 0–1 month, subjects obtaining RR-M: Intervention 0, Control 5, 2-tailed Fisher Exact Test	0.06	
			b) 1–6 month, subjects obtaining RR-M: Intervention 8, Control 7, χ2 (df = 1, *N* = 209) = 0.44	0.51	
			b) 6–12 month, subjects obtaining RR-M: Intervention 10, Control 3, χ2 (df = 1, *N* = 194) = 3.80	0.05	
		Hooker 2011 [24]	No data are presented.		No data are presented.
Health outcomes					
Anxiety	Hopkins Symptom Checklist 25^a^	Armstrong 2005 [22]	Adjusted mean difference − 2.89^e^	0.45	No significant effect.
	Revised Impact of Event Scale, intrusion subscale^b^	Armstrong 2005 [22]	Adjusted mean difference 0.16^e^	0.89	No significant effect.
Distress	Impact of Event Scale^a^	Hooker 2011 [24]	b) 0–1 month: B 3.95, z 2.61	0.01	Women in the control group reported significantly decreased distress in the month following randomization compared to women in the DA group. From 1 to 6 months women in the DA group reported significantly reduced distress compared to women who received UC. From 6 to 12 months no significant differences between groups were found. By 12-months, the overall decrease in distress between the two groups was similar.
			b) 1–6 month: B − 3.71, z − 2.35	0.02	
			b) 6–12 month: B − 1.05, z − 0.67	0.51	
		Metcalfe 2017 [25]	a) 3 month: Intervention 24.6 (13.9), Control 26.8 (12.8)	0.33	Women in the DA group showed significantly lower cancer related distress at 6 and 12 month post-randomization compared to the control group.
			a) 6 month: Intervention 19.3 (13.2), Control 25.2 (14.5)	0.01	
			a) 12 month: Intervention 17.7 (14.7), Control 22.4 (15.5)	0.05	
	Multidimensional Impact of Cancer Risk Assessment Questionnaire^b^	Hooker 2011 [24]	b) 0–1 month: B 3.08, z 2.01	0.04	At 1 month post-randomization women in the control group showed significantly decreased distress relative to the DA group. From 1 to 6 months and from 6 to 12 months, the groups did not differ significantly in their decrease of distress.
			b) 1–6 month: B − 1.35, z − 1.08	0.28	
			b) 6–12 month: B − 0.32, z − 0.25	0.80	
	Brief Symptom Inventory, modified scale^c^	Hooker 2011 [24]	b) B − 0.46, z − 0.54	0.59	No significant effect.

*RCT* randomized controlled trial, *DA* decision aid, *OC* ovarian cancer, *BC* breast cancer, *RR-M* risk-reducing mastectomy, *RR-O* risk-reducing oophorectomy, *HRT* hormone replacement therapy

^a^Instrument was validated in a study

^b^unclear, if instrument was validated

^c^instrument was not validated

^d^risk estimates from 0 to 100%

^e^Unclear comparison: The time points and groups are not specified

**Table 4:** Outcomes, instruments used, and effects of decisions aids evaluated in the included one-group pretest-posttest design study

Outcomes	Instruments used for assessment	Pretest-posttest study using the instrument	Score (S.D.)	p-value	Main results
Decision related outcomes					
Decisional conflict	Decisional Conflict Scale^a^	Metcalfe 2007 [27]	Pre-test 36.2 (16.4), Post-test 23.0 (15.2)	0.001	Significantly less decisional conflict after using the DA.
Strenght of treatment preference	15-point scale	Metcalfe 2007 [27]	RR-M:		Significantly fewer women in the DA group were uncertain about RR-M and RR-O.
			Pre-test No 14, Yes 3, Unsure 3		
			Post-test No 10, Yes 4, Unsure 6	0.009	
			RR-O:		
			Pre-test No 5, Yes 12, Unsure 3		
			Post-test No 2, Yes 15, Unsure 3	0.003	
			Tamoxifen:		
			Pre-test No 10, Yes 1, Unsure 9		
			Post-test No 11, Yes 5, Unsure 4	0.12	
Information related outcomes					
Risk perception	BC risk, mutation carriers^b^	Metcalfe 2007 [27]	Pre-test 65.1 (16.1), Post-test 73.6 (13.4)	0.05	Significantly better risk perception after using the DA.
	BC, risk after RR-M, mutation carriers^b^	Metcalfe 2007 [27]	Pre-test 71.8 (22.0), Post-test 84.2 (18.2)	0.005	Significantly better risk perception after using the DA.
	BC, risk after RR-O, mutation carriers^b^	Metcalfe 2007 [27]	Pre-test 43.2 (20.0), Post-test 65.0 (13.3)	0.001	Significantly better risk perception after using the DA.
	BC, risk with Tamoxifen, mutation carriers^b^	Metcalfe 2007 [27]	Pre-test 50.0 (19.0), Post-test 56.6 (10.0)	0.17	No significant difference.
	BC, risk with mammography, mutation carriers^b^	Metcalfe 2007 [27]	Pre-test 21.5 (28.0), Post-test 13.5 (22.8)	0.11	No significant difference.
Health outcomes					
Distress	Impact of Event Scale^a^	Metcalfe 2007 [27]	Pre-test 22.7 (13.7), Post-test 19.9 (14.5)	0.24	No significant difference.

*DA* decision aid, *BC* breast cancer, *RR-M* risk-reducing mastectomy, *RR-O* risk-reducing oophorectomy

^a^instrument was validated in a study

^b^risk estimates from 0 to 100%

### Decision related outcomes

The qualitative synthesis of the evaluation studies showed that DA for *BRCA1* and *BRCA2* mutation carriers can have significant beneficial effects on decision related outcomes. Four studies evaluated the decisional conflict [22, 23, 25, 27]: One study showed a significant decline in mean decisional conflict scores in the DA group [27], one detected significant decreases in decisional conflict in initially undecided women using the DA [23] and one showed a significant reduction in scores on seven items from the Decisional Conflict Scale in the intervention group compared to the control [22]. One study evaluating the decisional conflict showed no significant effect between the intervention and the control groups [25].

Four studies measured the strength of treatment preference [23–25, 27]. Of these four, two studies showed a significantly beneficial effect of the DA: One found an increased likelihood to reach a management decision in initially undecided women in the DA group [23], and one showed that fewer women were uncertain about RR-M and risk-reducing oophorectomy [27]. One study evaluating this outcome showed no significant effect between the intervention and the control groups [25] and one study did not present data regarding this outcome [24].

Two studies evaluated the outcome “decision satisfaction” [22, 23]. Of these, one study found a significantly beneficial effect of the DA in initially undecided women [23]. One study using a 12-item-scale that combined items from the Decisional Conflict Scale with the Satisfaction With Decision Scale found that women using the DA were significantly more satisfied with their decision compared to the control group [22, 23]. Nevertheless, when analyzing only the scores of the Satisfaction with Decision Scale, no significant differences were found.

### Information related outcomes

Three studies evaluated the influence of the DA on risk perceptions of the affected women [22, 25, 27], two of them showing no significant difference between the DA and the control groups [22, 25]. One study showed a significant increase in knowledge scores [27].

### Actual preventive choice

Two studies measured the actual preventive choice made by the included women [23, 24]. One study revealed that regarding the actual preventive choice of RR-M there was no significant difference between the control and the DA group [23]. However, the DA had an impact on the timing of the preventive measure. Women in the control group tended to have a RR-M early after genetic testing, thus not reaching statistical significance, whereas significantly more women using the DA tended to have the procedure 6 to 12 month after genetic testing. One study provided no data about the outcome of the actual preventive choice [24].

### Health outcomes

A variety of instruments were used in the trials to determine health outcomes. Three studies measured distress [24, 25, 27] and one anxiety [22].

One study [24] measured distress using three different instruments: The Impact of Event Scale (IES) to measure cancer specific distress, a modified version of the Brief Symptom Inventory (BSI) to measure general distress and the Multidimensional Impact of Cancer Risk Assessment Questionnaire (MICRA) to measure genetic testing distress. Using the IES and the MICRA, the study showed that there were significantly higher distress scores in the DA group than in the control group in the month following randomization. In a subanalysis with women who reported that they actually have used the DA, the genetic testing distress at 12-month post-randomization was significantly lower in the DA group. Using the BSI no significant differences between the two groups were found [24].

Another study using the IES showed that women in the DA group experienced significantly lower cancer related distress at 6- and 12-month post-randomization compared to the control group [25]. One study indicated no significant effect on health outcomes [22].

## Discussion

This is the first systematic review of the effectiveness of DA for women with a pathogenic *BRCA1* or *BRCA2* mutation who are facing the difficult and complex decision of choosing a preventive strategy that goes well with their individual life situation and personal values. Our review gives an overview of the quality of the existing evidence and summarizes the effects of DA on decision, information and health related outcomes as well as on actual choice for preventive measures.

### Quality of included studies

Concerning the study quality three of the four RCT showed a high risk of bias, one showed an unclear risk. The strengths of the included RCT were their adequate sequence generation, their complete outcome reporting and the absence of other sources of bias. The main weakness of the identified studies was the insufficient blinding of patients and personnel. Blinding remains challenging in the field of decision support as recipients usually can recognize the assigned intervention. However, options such as providing a DA versus a non-structured set of information materials or flyers should be considered in future studies. Apart from the four RCT, one study with a one-group pretest-posttest design was included, because it fulfilled the inclusion criteria. This study showed a serious risk of bias. Its main deficits were found in the domains confounding and measurement of outcomes. The weak quality of this study is not surprising, since the one-group pretest-posttest design is often criticized because of different threats to its internal and external validity [28, 29].

In summary, the quality of the studies included in this review is compromised to some extent. The results regarding the effects of the DA must be seen in the light of this weakness.

### Decision related outcomes

We found indications that the use of a DA for female *BRCA1* and *BRCA2* mutation carriers has some advantages with respect to decision related outcomes. Three studies show that the use of a DA leads to significantly less conflict during the decision-making process [22, 23, 27]. This applies especially to women who are initially undecided. The use of a DA also significantly increases the likelihood to reach a decision in initially undecided women [23]. Finally, two studies demonstrate that mutation carriers using a DA are significantly more satisfied with their decision compared to the control group [22, 23].

Our findings are partly congruent with the results of the most recent Cochrane Collaboration review about the effectiveness of DA for people facing health treatment or screening decisions. In this review 105 RCT involving 31,043 participants were analyzed [17]. The Cochrane Collaboration review contrasts in some points with our review: It included studies about DA for various – and not only one specific – health decisions, it included only studies with an RCT design, and it excluded studies, when the relevant DA were not available. This may explain, why only two of the studies [22, 24] which were included in this review are also apparent in the Cochrane Collaboration review.

Consistent with our results, the Cochrane Collaboration review showed that the use of DA leads to a lower decisional conflict. In contrast to our results, the Cochrane Collaboration review found that the effects on satisfaction are limited. This limited effect is, according to the Cochrane Collaboration review, possibly a consequence of measurement insensitivity: Because satisfaction with usual care is already high, it is difficult to measure differences in satisfaction between DA and control groups. The studies included in the Cochrane Collaboration review used a variety of instruments to measure satisfaction, most of them were not validated. Also, in our review only one of two studies evaluating the effect of the DA on satisfaction used a validated instrument, more precisely the Satisfaction with Decision Scale. Therefore, to make a clear statement about the effects of a DA on satisfaction, more high-quality evidence is needed, including studies using validated and sensitive instruments.

### Information related outcomes

The main conclusion of the Cochrane Collaboration review is that the largest benefits of DA compared to usual care, are better knowledge and risk perceptions [17]. These results contrast with our findings. In our review, regarding risk perceptions, two studies [22, 25] showed no significant effect. One study [27] showed a significant increase in knowledge scores, but due to the study design and the lack of a control group without intervention this result cannot carry much weight [28, 29]. There might be several reasons for the differing effects on knowledge. The Cochrane Collaboration included DA designed for 50 different health decisions. The target groups of these DA were heterogeneous in age and sex. We only included DA addressing preventive decisions of mostly middle-aged female *BRCA1* and *BRCA2* mutation carriers. As younger and middle-aged females have higher levels of health literacy [30, 31] and might have higher knowledge in health topics in general, it could be hypothesized that information related effects might be higher in other populations.

Metcalfe and colleagues [25] reason that they could not detect large changes in knowledge scores, because all included women in their RCT were well informed and already had very high knowledge scores at baseline. This in turn is, according to Metcalfe, a sign of the impact of successful pre- and posttest genetic counselling which ideally leads to an informed patient. This argument can be reinforced by different studies. In a systematic review of Nelson and colleagues eight studies from 2004 to 2011 reported improved accuracy of BC risk perception after genetic counseling [32]. In another systematic review Butow and colleagues are concluding, that genetic counselling is, at least in the short term, successfully enhancing the accuracy of women’s risk perception [33].

### Actual preventive choice

Regarding the actual preventive choice, we found only marginal effects of the use of a DA. Although not reaching statistical significance, Schwartz and colleagues [23] demonstrated that more women in the DA group were tending to do a RR-M. Furthermore, they found out that the women in the DA group would do the procedure significantly later than the women in the control group [23]. Schwartz assumes that the delayed RR-M is a sign of a higher grade of deliberation due to the DA.

This argument is supported by a study of Howard and colleagues [34], who performed in-depth interviews with female *BRCA1* and *BRCA2* carriers about the “right time” of considering risk-reducing surgery. According to the study, the interviewed women felt that it is necessary to take enough time to deliberate about risk-reducing surgery decisions. Otherwise, they would not feel “ready” and “able” to make these decisions.

In contrast to our results, the latest Cochrane Collaboration review on DA showed, excluding Schwartz et al. [23], a significant reduction in the number of patients in the DA group choosing major elective surgery [17]. The authors argue that people in the control groups may be more inclined towards surgery due to their deficient or missing awareness of alternatives, benefits and harms. It is important to underline that the results of the Cochrane Collaboration review are based on outcomes of different studies with groups of patients facing different health decisions, such as cardiac revascularization, bariatric surgery and orchiectomy. A direct comparison of these groups to female *BRCA1* and *BRCA2* mutation carriers who consider various preventive options, is difficult, especially because their decision for or against risk-reducing surgery is very complex and influenced by many factors such as lifetime risk of developing cancer, family history of cancer, having children and age [35, 36].

Therefore, more high-quality evidence is needed to make a clear statement about the effects on the actual preventive choice of preventive measures by the use of a DA for *BRCA1* and *BRCA2* mutation carriers.

### Health outcomes

As for health outcomes such as anxiety or distress, two studies revealed an impact of the DA versus the control group which varied over time [24, 25]. One study [24] showed that women in the DA group had significantly higher scores in cancer specific and genetic testing distress in the month following randomization, but significantly less genetic testing distress at 12-month post-randomization. Hooker and colleagues [24] suggested that those short-term increases in distress in the DA group are a sign of ongoing deliberation and cognitive processing. This hypothesis in turn could be supported by the study of Metcalfe and colleagues [25] which demonstrated that the DA group showed significantly lower cancer related distress at 6 and 12 month post-randomization compared to the control group.

In contrast to our results, the effects of DA on health outcomes, such as general health outcomes, anxiety, depression, regret and confidence, in the latest Cochrane Collaboration review were limited [17]. In the Cochrane Collaboration review a variety of different instruments were used to investigate the effects on health outcomes. Nevertheless, the three instruments which were utilized in this review to investigate patients’ distress and showed significant changes – the BSI, the MICRA and the IES – were not part of it. This may explain the different results of the Cochrane Collaboration review on health outcomes, especially on distress, and our work.

### Strengths and limitations

Our findings must be considered in the light of the strengths and limitations of this review. The main limitation is that the identified studies showed a high or unclear risk of bias. This might restrict the information value of the validity of DA effectiveness as summarized in this study.

Another limitation is that this review only provides a qualitative summary of the outcome results of the included RCT. A meta-analysis was not possible because the identified studies were heterogeneous in terms of study design, time frame between genetic test result disclosure and delivery of the decision aid, follow-up periods and the choice of outcomes. Moreover, we could not access all the required data from all studies [22–24], despite contacting the authors.

The strengths of this review are its clear search protocol, the inclusion of six different databases and the involvement of three different reviewers. The critical quality assessment based on the well-established Cochrane Collaborations’ risk of bias tool and the ROBINS-I tool ensures a transparent judgement of studies. We included all types of studies and outcomes in our review, which allows to provide a complete overview of the effectiveness of DA for *BRCA1* and *BRCA2* mutation carriers. Moreover, we sent author request to obtain data in other formats so that they might be included in our review.

## Conclusions

This is the first systematic review of the effectiveness of DA for women with a pathogenic *BRCA1* or *BRCA2* mutation. Our work indicates that DA may support female *BRCA1* and *BRCA2* mutation carriers during their decision process for choosing a preventive measure. This is mainly achieved by improving decision related outcomes. More high-quality evidence is needed to evaluate possible advantages or disadvantages on information related and health outcomes as well as on the actual choice for preventive measures. To provide high-quality evidence and to reach a higher comparability of study results it is important that future research focuses on 1) low-biased study designs and 2) the use of well-established and validated instruments to assess outcomes.

## Additional files

Additional file 1: Full search strategy for each database. (DOCX 17 kb)

Additional file 2: Quality of randomized controlled trials / Rationale. (XLSX 15 kb)

Additional file 3: Quality of pretest-posttest study / Rationale. (XLSX 14 kb)

## Data Availability

The datasets used and/or analyzed during the current study are available from the corresponding author on reasonable request.

## Abbreviations

BC: Breast cancer
*BRCA1*: Breast cancer gene 1
*BRCA2*: Breast cancer gene 2
BSI: Brief Symptom Inventory
CD-ROM: Compact Disc Read-Only Memory
DA: Decision aid(s)
HRT: Hormone replacement therapy
IES: Impact of Event Scale
IPDAS: International Patient Decision Aids Standards
MICRA: Multidimensional Impact of Cancer Risk Assessment Questionnaire
NL: Netherlands
OC: Ovarian cancer
PROM: Patient-reported outcome measures
RCT: Randomized controlled trial(s)
RR-BM: Risk-reducing bilateral mastectomy
RR-BSO: Risk-reducing bilateral salpingo-oophorectomy
RR-CM: Risk-reducing contralateral mastectomy
RR-M: Risk-reducing mastectomy
RR-O: Risk-reducing oophorectomy
