# Supplementary material for: Effectiveness of decision aids for female BRCA1 and BRCA2 mutation carriers: a systematic review
Source: BMC Med Inform Decis Mak. 2019 Aug 1;19:154. doi: 10.1186/s12911-019-0872-2 (PMC6670224; doi:10.1186/s12911-019-0872-2)
Supplement: Supplementary file 1 — Full search strategy for each database. (DOCX 17 kb) [file 12911_2019_872_MOESM1_ESM.docx]

### Additional file 1 Full search strategy for each database

Database: MEDLINE

Date of Search: 05.02.2019

Search Strategy:

--------------------------------------------------------------------------------

1 brca/ (4464)

2 brca1/ (15445)

3 brca2/ (9819)

4 brca1/2/ (2763)

5 1 or 2 or 3 or 4/ (18742)

6 decision making/ (286633)

7 decision aid/ (85775)

8 decision support tool/ (9475)

9 6 or 7 or 8/ (359600)

10 5 and 9/ (671)

| **Translations:** |
| --- |
| \| brca1 \| "genes, brca1"[MeSH Terms] OR ("genes"[All Fields] AND "brca1"[All Fields]) OR "brca1 genes"[All Fields] OR "brca1"[All Fields] \| \| --- \| --- \| \| brca2 \| "genes, brca2"[MeSH Terms] OR ("genes"[All Fields] AND "brca2"[All Fields]) OR "brca2 genes"[All Fields] OR "brca2"[All Fields] \| \| Decision aid \| "decision support techniques"[MeSH Terms] OR ("decision"[All Fields] AND "support"[All Fields] AND "techniques"[All Fields]) OR "decision support techniques"[All Fields] OR ("decision"[All Fields] AND "aid"[All Fields]) OR "decision aid"[All Fields] \| \| Decision making \| "decision making"[MeSH Terms] OR ("decision"[All Fields] AND "making"[All Fields]) OR "decision making"[All Fields] \| \| Decision \| "Decision (Wash D C )"[Journal] OR "decision"[All Fields] \| |

Database: ERIC

Date of Search: 05.02.2019

Search Strategy:

--------------------------------------------------------------------------------

1 brca/ (8)

2 brca1/ (5)

3 brca2/ (3)

4 brca1/2/ (3)

5 1 or 2 or 3 or 4/ (8)

6 “decision making”/ (54271)

7 “decision aid”/ (178)

8 “decision support techniques”/ (2)

9 “decision support tool”/ (34)

10 6 or 7or 8 or 9/ (54359)

11 5 and 10/ (2)

Database: EMBASE

Date of Search: 05.02.2019

Sources: EMBASE

Search Strategy:

--------------------------------------------------------------------------------

1 “brca”/ (8469)

2 “brca1”/ (24290)

3 “brca2”/ (16006)

4 “brca1/2”/ (4886)

5 1 or 2 or 3 or 4/ (30682)

6 “decision making”/ (288019)

7 “decision aid”/ (1931)

8 “decision support techniques”/ (466)

9 “decision support tool”/ (1366)

10 6 or 7 or 8 or 9/ (289712)

11 5 and 10/ (1129)

Database: PsycINFO

Date of Search: 05.02.2019

Search Mode: Boolean/Phrase

Search Strategy:

--------------------------------------------------------------------------------

1 brca/ (216)

2 brca1/ (347)

3 brca2/ (277)

4 brca1/2/ (200)

5 1 or 2 or 3 or 4/ (527)

6 “decision making”/ (121735)

7 “decision aid”/ (667)

8 “decision support techniques”/ (1292)

9 “decision support tool”/ (251)

10 6 or 7or 8 or 9/ (122402)

11 5 and 10/ (139)

Database: Cochrane Library

Date of Search: 05.02.2019

Search Mode: All text

Search Limits: Search word variations

Search Strategy:

--------------------------------------------------------------------------------

1 “BRCA”/ (348)

2 “BRCA1”/ (518)

3 “BRCA2”/ (321)

4 “BRCA1/2”/ (239)

5 1 or 2 or 3 or 4/ (717)

6 “decision making”/ (11048)

7 “decision aid”/ (930)

8 “decision support techniques”/ (743)

9 “decision support tool”/ (274)

10 6 or 7or 8 or 9/ (11809)

11 5 and 10/ (59)

Database: CINAHL

Date of Search: 05.02.2019

Search Mode: Boolean/Phrase

Limits: Exclude MEDLINE Records

Search Strategy:

--------------------------------------------------------------------------------

1 brca/ (878)

2 brca1/ (325)

3 brca2/ (244)

4 brca1/2/ (175)

5 1 or 2 or 3 or 4/ (1131)

6 “decision making”/ (49203)

7 “decision aid”/ (267)

8 “decision support techniques”/ (859)

9 “decision support tool”/ (151)

10 6 or 7or 8 or 9/ (49821)

11 5 and 10/ (93)
